# Clinical Epidemiology of Septic Arthritis Caused by *Burkholderia pseudomallei* and Other Bacterial Pathogens in Northeast Thailand

**DOI:** 10.4269/ajtmh.17-0288

**Published:** 2017-10-02

**Authors:** Prapit Teparrukkul, Jiraphorn Nilsakul, Susanna Dunachie, Direk Limmathurotsakul

**Affiliations:** 1 Medical department, Sunpasitthiprasong Hospital, Ubon Ratchathani, Thailand;; 2 Microbiological department, Sunpasitthiprasong Hospital, Ubon Ratchathani, Thailand;; 3 Nuffield Department of Medicine, Centre for Tropical Medicine and Global Health, University of Oxford, Oxford, United Kingdom;; 4 Mahidol-Oxford Tropical Medicine Research Unit, Faculty of Tropical Medicine, Mahidol University, Bangkok, Thailand;; 5 Department of Tropical Hygiene, Faculty of Tropical Medicine, Mahidol University, Bangkok, Thailand

## Abstract

Septic arthritis is a medical emergency, and if not treated appropriately, it can be associated with high morbidity and mortality. Melioidosis, a serious infectious disease caused by the Gram-negative bacillus *Burkholderia pseudomallei*, is highly endemic in South and Southeast Asia and northern Australia. We reviewed the medical charts of adult patients admitted with bacterial septic arthritis at Sunpasitthiprasong Hospital, Ubon Ratchathani, northeast Thailand from January 2012 to December 2014. Bacterial septic arthritis was defined as one or more hot swollen joints with isolation of a pathogenic organism from an affected joint or from blood. A total of 154 patients with septic arthritis were retrospectively evaluated. The most common causes were *B. pseudomallei* (48%, *N* = 74), *Streptococcus* spp. (29%, *N* = 44), and *Staphylococcus aureus* (10%, *N* = 16). Prevalence of diabetes, bacteremia, and pneumonia was higher in *B. pseudomallei* septic arthritis than in septic arthritis caused by the other bacteria (all *P* < 0.01). Seventy three percent (54/74) of patients infected with *B. pseudomallei* and 69% (55/80) of patients with the other bacteria received effective antimicrobials on the first day of admission (*P* = 0.60), but in-hospital mortality of the former group was considerably higher (34% versus 14%, *P* = 0.004). In conclusion, *B. pseudomallei* septic arthritis is common and associated with high mortality in northeast Thailand. Emergence of *Streptococcus* arthritis is observed. Difficulty in diagnosing melioidosis and identifying *B. pseudomallei* in areas where health care workers are not familiar with the disease is discussed. In melioidosis-endemic regions, parenteral ceftazidime could be considered as empirical antimicrobial therapy for patients with septic arthritis and underlying diseases.

## INTRODUCTION

Septic arthritis is a common medical emergency and is associated with substantial morbidity and mortality.^1^ The case fatality rate (CFR) for septic arthritis is about 5–15%, and irreversible loss of joint function develops in 25–50% of those who survive.^2^ The infection is usually caused by hematogenous spread during a transient or persistent bacteremia, or by direct inoculation occurring with trauma or iatrogenically. The most frequent causative organisms identified internationally are *Staphylococcus aureus* and *Streptococcus* spp., whereas Gram-negative bacteria such as *Escherichia coli* and *Pseudomonas* spp. are more common in the older population and in those with recurrent urinary tract infection. *Neisseria gonorrhoeae* is now a rare cause of septic arthritis in Europe and North America,^1^ but is still prevalent in some parts of the world.^3,4^ Therefore, the initial antibiotic choice in patients suspected with septic arthritis is based on clinical risk factors and local epidemiology.^1^

Melioidosis is an infection cause by *Burkholderia pseudomallei*, a Gram-negative bacillus founded in soil and water. The disease is also increasingly reported as a cause of septic arthritis in those who are living in or traveling to those endemic countries.^3,5,6^ *Burkholderia pseudomallei* is intrinsically resistant to a wide range of antimicrobials, including gentamicin and most third-generation cephalosporins except ceftazidime. Effective parenteral antibiotics include ceftazidime and carbepenem.^7^ Even with effective antimicrobial treatment, the CFR of melioidosis is high, ranging from 14% in northern Australia^8^ to 40% in northeast Thailand.^9^ Melioidosis is predicted to be endemic in 82 tropical countries, with a high incidence rate in South Asia, Southeast Asia, and northern Australia.^10^ Diabetes mellitus is the most important risk factor, and is found in about 50% of melioidosis patients.^9^ Other known risk factors include exposure to soil or water, male gender, older age, excess alcohol consumption, chronic liver disease, chronic lung disease, chronic renal disease, and thalassemia. Nonetheless, about 20% of adult melioidosis patients have no recognized risk factors.^9,11^

Here, we review cases presenting with septic arthritis to a tertiary referral hospital in northeast Thailand. We also compare clinical aspects, management, and outcomes between those caused by *B. pseudomallei* and the other bacterial pathogens.

## MATERIALS AND METHODS

### Study patients.

A retrospective study was conducted to review all medical records of adult patients (age > 15 years old) admitted with septic arthritis at Sunpasitthiprasong Hospital, Ubon Ratchathani, northeast Thailand, from January 1, 2012 to December 31, 2014. Cases were searched via the hospital admission database for patients with a final diagnosis of pyogenic arthritis (International Classification of Disease, tenth Revision [ICD-10] diagnostic code of M00 or M01) and via the microbiological laboratory database searching for patients with synovial fluid culture positive for any organism. Culture-confirmed bacterial septic arthritis was defined as one or more joints with typical features of septic arthritis, and isolation of a pathogenic organism from an affected joint or from blood. Typical features of septic arthritis were defined as at least one of the compatible clinical symptoms and signs: redness, swelling, heat, pain, limited range of motion, and imaging documentation.

### Data collection.

Demographic data, presenting symptoms and signs, duration of symptoms before admission, underlying disease, history of previous joint surgery or injection, and imaging results were collected. The involved joint and microbiological results for synovial fluid and blood, treatment, and outcome at hospital discharge and at 1-year follow up were recorded.

Parenteral antimicrobials prescribed for patients with septic arthritis were considered effective if the isolated organism were susceptible to that antimicrobial on the susceptible testing. Only ceftazidime, carbapenem drug, and amoxicillin/clavulanic acid were considered effective against *B. pseudomallei*. Arthrotomy was defined as an open joint procedure. Arthroscopic washout was not available in the hospital during the study period.

### Cumulative incidences of bacteremia.

We estimated cumulative incidences of bacteremia to compare the most common causes of septic arthritis and bacteremia in the same setting. The overall cumulative incidence was estimated by the total number of new bacteremia cases divided by the total population of Ubon Ratchathani at the beginning of the study period. The total number of new bacteremia cases from January 2012 to December 2014 was estimated from the microbiological laboratory database. Ubon Ratchathani province had an estimated population in 2012 of 1.86 million. Because of the difficulty in establishing their clinical significance, organisms frequently associated with contamination including coagulase-negative *staphylococci*, viridans group *streptococci*, *Corynebacterium* spp., *Bacillus* spp., *Diptheroid* spp., *Micrococcus* spp., and *Propionibacterium* spp. were excluded from the analysis for cumulative incidences of bacteremia. Only the first episode of bacteremia for each patient was included in the analysis.

### Statistical analysis.

Categorical variables were compared using χ^2^ test or Fisher’s exact test. Continuous variables were compared using Mann–Whitney *U* test. Interquartile ranges (IQRs) are presented as 25th and 75th percentiles. Logistic regression models were used to calculate odds ratios. All analyses were performed using STATA version 14.0 (StataCorp LP, College station, TX). Because the cumulative incidences of *Streptococcus* septic arthritis was much higher than *S. aureus* arthritis in our setting during the study period, we conducted additional analysis by comparing clinical manifestations between septic arthritis caused by *Streptococcus* spp. and *S. aureus*.

### Ethics statement.

Approval for the study was obtained from the Ethics Committee of Sunpasitthiprasong Hospital (059/2014).

## RESULTS

**Figure 1.**
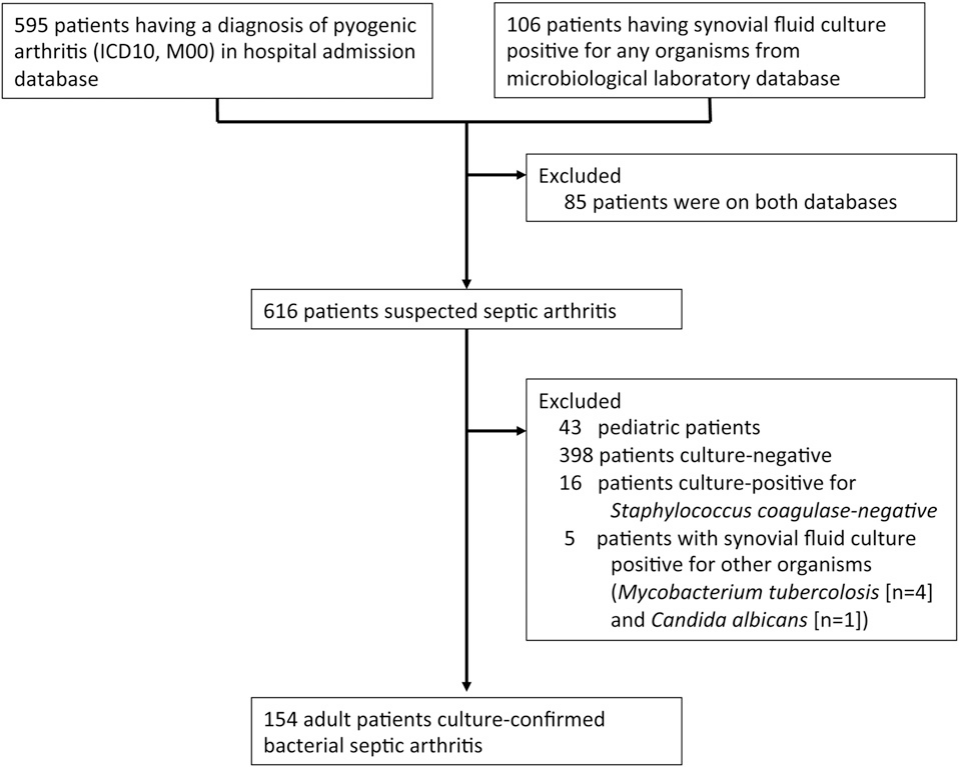
Study flow diagram.

A total of 616 medical case records were identified by the search of both the hospital admission database for patients with a final diagnosis of pyogenic arthritis and the microbiological hospital database for patients with synovial fluid culture-positive for any bacteria (Figure 1). A total of 43 cases were pediatric patients, 398 cases were culture-negative for any organism, 16 cases were culture-positive for *Staphylococcus* coagulase negative (considered as bacterial contaminants in the context of native joints), four cases had synovial fluid culture-positive for *Mycobacterium tuberculosis*, and one case was positive for *Candida albicans*. Therefore, 154 adult patients with culture-confirmed bacterial septic arthritis were included in the final analysis.

A total of 106 (69%) patients had a positive synovial fluid culture, and 113 (73%) patients had a positive blood culture (Table 1). The most common causative pathogens were *B. pseudomallei* (48%, *N* = 74), *Streptococcus* spp. (29%, *N* = 44) and *S. aureus* (10%, *N* = 16). Other pathogenic organisms observed included *Klebsiella* spp. (5), *E. coli* (4), *Enterococcus* spp. (3), *Salmonella* spp. (3), *Pseudomonas aeruginosa* (2), *Enterobacter* spp. (2) and *Acinetobacter* spp. (1). Of 16 patients infected with *S. aureus*, three (19%) were caused by methicillin-resistant *S. aureus*. Two were patients with end-stage renal disease on hemodialysis, and the other one was a paraplegic patient with necrotizing fasciitis. Of four *E. coli* and five *Klebsiella* spp. cases, two (50%) and one (20%) were infected with extended-spectrum beta-lactamases–producing isolates, respectively.

**Table 1:** Causative organisms

Organisms	Number of patients (%, *N* = 154)	Synovial fluid culture positive	Blood culture positive
*Burkholderia pseudomallei*	74 (48)	55	64
*Streptococcus* spp.	44 (29)	21	32
*Staphylococcus aureus*	16 (10)	12	13
*Klebsiella* spp.	5 (3)	4	2
*Escherichia coli*	4 (3)	4	0
*Enterococcus* spp.	3 (2)	2	1
*Salmonella* spp.	3 (2)	3	0
*Pseudomonas aeruginosa*	2 (1)	2	0
*Enterobacter* spp.	2 (1)	2	0
*Acinetobacter* spp.	1 (1)	0	1

Table 2 shows the demographic of the study population. A total of 92 (60%) patients were male, and the median age at presentation was 55 years old (IQR 45–66 years). Gender and age were not different between *B. pseudomallei* septic arthritis and those caused by the other bacterial pathogens. Three-quarters of *B. pseudomallei* septic arthritis presented during rainy season (defined as the 6-month period from June to November) whereas half of those caused by other bacterial pathogens presented during rainy season (73% versus 58%, *P* = 0.06). Diabetes mellitus was more common in those caused by *B. pseudomallei* than in those caused by the other bacterial pathogens (88% versus 19%, *P* < 0.001). Percentages of patients without any underlying risk factors were higher in those caused by the other bacterial pathogens than in those caused by *B. pseudomallei* (56% versus 4%, *P* < 0.001). A previous episode of melioidosis was observed only in those who presented with *B. pseudomallei* septic arthritis (9% versus 0%, *P* = 0.005), whereas two patients who had joint prosthesis were infected with *Streptococcus* spp. (*N* = 1) and *E. coli* (*N* = 1).

**Table 2:** Case demographics

Variables	*Burkholderia pseudomallei* septic arthritis (*N* = 74)	Other septic arthritis (*N* = 80)	*P* value*
Male gender	42 (57%)	50 (63%)	0.51
Age (year, median, IQR)	53 (44–61)	58 (48–67)	0.22
Presenting during rainy season†	54 (73%)	46 (58%)	0.06
Risk factors for development of septic arthritis			
Diabetes mellitus	65 (88%)	15 (19%)	< 0.001
Chronic kidney disease	13 (18%)	9 (11%)	0.36
Cutaneous ulcers	8 (11%)	13 (16%)	0.33
Gouty arthritis	4 (5%)	9 (11%)	0.36
Rheumatoid arthritis	2 (3%)	1 (1%)	0.61
Alcoholism	0	3 (4%)	0.25
Joint prosthesis	0	2 (3%)	> 0.99
Intravenous drug abuse	0	0	NA
History of melioidosis	7 (9%)	0	0.005
None	3 (4%)	45 (56%)	< 0.001

IQR = interquartile range.

* *P* value derived using χ^2^ test or Fisher’s exact test.

† From June to November.

Overall, three quarters (76%; 118/154) presented with monoarthritis, and a quarter (23%; 35/154) presented with polyarthritis (Table 3). The most frequently involved joint was knee (64%; 98/154), and the percentage of involvement was lower in those infected with *B. pseudomallei* compared with other bacterial pathogens (53% versus 74%, *P* = 0.01). Hip was the fourth common joint involved (12%; 19/154), and the percentage of involvement was more common in those infected with *B. pseudomallei* compared with other bacterial pathogens (19% versus 6%, *P* = 0.03). Ten patients had osteomyelitis confirmed by standard radiography (*N* = 8) or magnetic resonance imaging (*N* = 2). Bacteremia and pneumonia were more common in those infected with *B. pseudomallei* than those infected by the other bacterial pathogens (*P* = 0.001 and < 0.001, respectively). Hepatosplenic abscess was observed in 31% of melioidosis patients who had abdominal imaging performed (16/51), whereas this was not observed in those caused by other bacterial pathogens (0/8, *P* = 0.09). Central nervous system (CNS) infection was observed in three melioidosis patients and three nonmelioidosis patients (*Streptococcus* spp. [*N* = 2] and *S. aureus* [*N* = 1]). CNS infection included brain abscess (*N* = 2), meningitis (*N* = 2), epidural abscess (*N* = 1), meningitis (*N* = 1), and meningoencephalitis (*N* = 1). A total of three melioidosis patients and two patients caused by other bacterial pathogens developed symptoms and signs of arthritis after hospital admission (4% versus 3%, *P* = 0.67).

**Table 3:** Sites of infection

Variables	*Burkholderia pseudomallei* septic arthritis (%, *N* = 74)	Other septic arthritis (%, *N* = 80)	*P* value*
No. of joints involved			
1 (monoarthritis)	61 (82)	58 (73)	0.18
≥ 2 (Polyarthritis)	13 (18)	22 (28)	–
Location of joints			
Knee	39 (53)	59 (74)	0.01
Shoulder	14 (19)	9 (11)	0.23
Hip	14 (19)	5 (6)	0.03
Ankle	11 (15)	14 (18)	0.67
Wrist	5 (7)	12 (15)	0.13
Elbow	4 (5)	2 (3)	0.43
Sternoclavicular	1 (1)	4 (5)	0.37
Osteomyelitis	3 (4)	7 (9)	0.33
Other organ involvements			
Blood†	64/74 (86)	49/79 (62)	0.001
Lung	19 (26)	1 (1)	< 0.001
Soft tissue	13 (18)	16 (20)	0.84
Skin	3 (4)	8 (10)	0.21
Urinary tract	8 (11)	2 (3)	0.05
Bone	3 (4)	7 (8)	0.33
Central nervous system	3 (4)	3 (4)	> 0.99
Lymph node	2 (3)	0	0.23
Liver or splenic abscess‡	16/51 (31)	0/8 (0)	0.09

* *P* value derived using χ^2^ test or Fisher’s exact test.

† Blood culture was not performed in one case.

‡ In patients who had abdominal imaging by ultrasonography, CT scan, or MRI.

The joint was aspirated in 112 patients (72%). The synovial fluid was recorded as pus without a white blood cell count report in 22 patients (12 melioidosis and 10 nonmelioidosis patients, *P* = 0.67), and the white blood cell count was not recorded in another 13 patients. Of 77 patients with a white blood cell count available, the median white blood cell count was not different between melioidosis and nonmelioidosis patients (29,965 [IQR 9,910–105,000] versus 35,600 [IQR 12,400–173,000]/mm^3^, *P* = 0.47). The median proportion of neutrophils was also not different between the two groups (91% [IQR 81–94%] versus 90% [IQR 80–95%], *P* = 0.97).

Overall, 109 patients (71%) received effective antimicrobials on the day of hospital admission, and the median time from admission to effective antimicrobials were not different between the two groups (*P* = 0.60, Table 4). Percentages of patients who received arthrotomy washout was not significantly different between the two groups (*P* = 0.13). In the univariable model, the CFR of patients infected with *B. pseudomallei* was higher than those caused by other bacterial pathogens (34% versus 14%, *P* = 0.004), and CFR of patients with blood culture positive was higher than those without blood culture positive (28% [32/113] versus 10% [4/41], *P* = 0.02). In the multivariable logistic regression model, there was strong and borderline evidence showing that *B. pseudomallei* septic arthritis (adjusted odds ratio [aOR] 2.6; 95% CI 1.2–6.0, *P* = 0.02) and blood culture positivity (aOR 2.7; 95% CI 0.9–8.6, *P* = 0.09) were associated with in-hospital mortality, respectively.

**Table 4:** Treatment and outcomes

Variables	*Burkholderia pseudomallei* septic arthritis (*N* = 74)	Other septic arthritis (*N* = 80)	*P* value*
Time from admission to effective antimicrobials (days, median, IQR, range)	0 (0–1, 0–16)	0 (0–1, 0–12)	0.67
Received effective antimicrobial on the admission day	54 (73%)	55 (69%)	0.60
Arthrotomy	53 (72%)	47 (59%)	0.13
In-hospital mortality	25 (34%)	11 (14%)	0.004
Duration of hospitalization in those who survived (days, median, IQR)	22 (14–32)	15 (9–31)	0.14
1-year outcome			
Recurrent septic arthritis	1/29 (3%)	0/26 (0%)	> 0.99
Deformity	2/29 (7%)	2/26 (7%)	> 0.99
Limited range of motion without deformity	3/29 (10%)	7/26 (27%)	0.16

IQR = interquartile range.

* *P* value derived using χ^2^ test or Fisher’s exact test, or using Mann–Whitney test for the continuous variables.

Data for 1-year outcome was available in 55 of 114 (48%) patients who survived. Recurrent septic arthritis, deformity, and limit range of motion without deformity were observed in 1, 4, and 10 patients, respectively (Table 4), and those 1-year outcomes were not significantly different between melioidosis and nonmelioidosis patients. Among nonmelioidosis patients, the proportion of patients with deformity or limited range of motion without deformity was also not significantly different among cases caused by *Streptococcus* spp. (38%, 6/16) and cases caused by *S. aureus* (38%, 3/8).

We also evaluated the incidences of bacteremia in the hospitals during the study period and found that the most common causes of bacteremia were *E. coli* (*N* = 1,221, 19%), *B. pseudomallei* (*N* = 836, 13%), *S. aureus* (*N* = 719, 11%), *Streptococcus* spp. (*N* = 654, 10%), and *Pseudomonas* spp. (*N* = 592, 9%). The overall cumulative incidences were 65.6, 44.9, 38.7, 35.2, and 31.8 per 100,000 population during the 3-year study period, respectively. We also compared clinical manifestations between septic arthritis caused by *Streptococcus* spp. and *S. aureus*, and those were not significantly different between the two groups.

## DISCUSSION

Our study shows that *B. pseudomallei* is the most common cause of septic arthritis in northeast Thailand, and patients with septic arthritis caused by *B. pseudomallei* have a higher CFR compared with those caused by other bacterial pathogens. In Europe and the United States, the most common cause of nongonococcal septic arthritis is *S. aurues*, and *Streptococcus* spp. are the next most common one.^12^ However, in our study, *Streptococcus* spp. are more common than *S. aureus* among non-*B. pseudomallei* septic arthritis. We did not observe the increase of cumulative incidence of *Streptococcus* bacteremia in general. The high proportion of *Streptococcus* septic arthritis in our study could relate to the reports of the emergence of group B *Streptococcus*^13^ and *Streptococcus suis*^14^ as causes of septic arthritis in Thailand. Further studies are needed to evaluate the emergence of *Streptococcus* arthritis in tropical developing countries. In melioidosis-endemic regions, parenteral ceftazidime could be considered as empirical antimicrobial therapy for patients with septic arthritis and underlying diseases. Choice of empirical antibiotic may need adjustment on the basis of local antimicrobial sensitivity profile of common pathogens for septic arthritis,^15,16^ and de-escalation to the most appropriate single-agent therapy should be performed as soon as the causative pathogen can be identified.

The high proportion of septic arthritis being caused by *B. pseudomallei* could be mainly because the incidence rate of melioidosis in the study area is high. We found that *B. pseudomallei* is the second most common cause of bacteremia during the study period after only *E. coli*. This is consistent with the previous finding that *B. pseudomallei* was also reported as the second most common cause of community-acquired bacteremia between 2004 and 2010 in northeast Thailand.^17^ This is also supported by the report that around 8% of culture-confirmed melioidosis patients in northeast Thailand had septic arthritis.^18^ There is increasing evidence suggesting that *B. pseudomallei* with *bimA* and *fhaB3* genes are associated with neurological and bacteremic melioidosis, respectively.^19,20^ It is still unknown whether any specific genotypes of *B. pseudomallei* are associated with septic arthritis, and further studies are needed. The number of patients with septic arthritis of joint prosthesis was very low (two patients), and that could be due to the low rate of prosthetic implants in the region.

The high CFR of septic arthritis caused by *B. pseudomallei* in our study is comparable with the CFR observed in all culture-confirmed melioidosis cases in Thailand^9^ and is consistent with the CFR observed in *B. pseudomallei* septic arthritis in other lower- and middle-income countries.^21^ *Burkholderia pseudomallei* is highly virulent in humans,^22^ and melioidosis patients often have a rapidly progressive illness, leading to multiple organ failures and high mortality. The observed CFR for septic arthritis caused by the other bacterial pathogens (14%) is in the range of 10–15% reported by other international studies.^23^ The higher CFR of septic arthritis caused by *B. pseudomallei* is consistent with the previous finding that the mortality of patients with *B. pseudomallei* bacteremia was higher than those with bacteremia caused by other bacterial pathogens.^24^ The high CFR in patients with septic arthritis and concomitant bacteremia is also consistent with the previous findings.^25,26^

Diagnosis of septic arthritis caused by *B. pseudomallei* can be challenging, particularly in areas where melioidosis is endemic but under- or never diagnosed.^10,27^ This is because clinical manifestations of melioidosis can be diverse, and *B. pseudomallei* can be commonly misidentified as a culture contaminant or as another species (e.g., *Burkholderia cepacia*, *Bacillus* spp., or *Pseudomonas* spp.), especially by laboratory staff unfamiliar with this organism.^28^ In resource-limited areas, a simple three disc diffusion test is recommended for screening all oxidase-positive Gram-negative rods, and *B. pseudomallei* should be suspected if the isolate is resistant to gentamicin and colistin/polymyxin, and susceptible to co-amoxiclav.^28,29^ Although we observed that diabetes mellitus and the presence of lung involvement is more commonly observed in septic arthritis caused by *B. pseudomallei*, those are not uncommon in septic arthritis caused by the other bacterial pathogens. On the contrary, history of melioidosis and the presence of liver or splenic abscess could be highly specific for melioidosis. The presence of prostatic abscess with septic arthritis should also suspect of melioidosis^5,8^; however, this is not observed in our study probably because prostatic abscess is not common in melioidosis patients in Thailand.^30^ Educating both clinicians and technicians about diagnosis of melioidosis is necessary.^7^ When clinicians suspect that *B. pseudomallei* is a possible cause of septic arthritis, clinicians should also notify microbiological laboratories so that laboratory technicians can perform appropriate testing for any oxidase-positive Gram-negative bacilli isolated and use appropriate biosafety practices to prevent laboratory exposure.^7,28^

The knee is the most commonly affected joint in septic arthritis caused by both *B. pseudomallei* and non-*B. pseudomallei*. This is consistent with the previous finding of *B. pseudomallei* septic arthritis in Australia.^31^ However, this is inconsistent with the previous finding in Khon Kaen, northeast Thailand, where *B. pseudomallei* septic arthritis was associated with an upper-extremity joint involvement.^32^ Immediate washout and adequate debridement of the septic joint are always needed. For those who are culture positive for *B. pseudomallei*, the minimum duration of antimicrobial treatment is 2 to 4 weeks of parenteral antimicrobials followed by a subsequent 90 days oral eradication phase.^7,33^ The recommended duration of the parenteral phase is timed from the date of most recent draining or resection where the culture of the draining or resected material grew *B. pseudomallei*, and the clock is reset if a subsequent specimen is culture positive.^33^

One-year outcomes for joint function were good for the majority of patients who were discharged alive from the hospital. This compares favorably to previous reports of outcome from septic arthritis.^23^ There was no difference between long-term outcomes for *B. pseudomallei* septic arthritis compared with other bacterial pathogens. Although *S. aureus* has been reported as associated with poorer long-term outcomes,^34^ we did not observe any difference between long-term outcomes for *Streptococcus* spp. and *S. aureus* septic arthritis.

A limitation of this study is that the isolates were not available for further investigations, and data of long-term outcomes were available only in a proportion of patients. It is also possible that septic arthritis caused by other bacterial pathogens is under-diagnosed in our study because those patients might have received antibiotics at primary hospitals or over the counter before blood or synovial fluid specimens were collected at our referral hospital. This is unlikely to decrease the yield of bacterial culture for *B. pseudomallei* because the organism is intrinsically resistant to commonly used first-line antibiotics. It is also possible that for a minority of patients with positive culture from the blood only, the bacteria isolated might not represent the cause of associated joint inflammation. Our study was not designed to have enough power to compare clinical manifestations between septic arthritis caused by *Streptococcus* spp. and *S. aureus*.

## CONCLUSIONS

In conclusion, we observe a high proportion of *B. pseudomallei* as a cause of septic arthritis in northeast Thailand, with higher mortality than for other bacterial causes of septic arthritis. Emergence of *Streptococcus* arthritis is observed. In melioidosis-endemic regions, a parenteral ceftazidime could be considered as empirical antimicrobial therapy for patients with septic arthritis and underlying diseases.
